# Patient-Physician Communication in Acute Myeloid Leukemia and Myelodysplastic Syndrome

**DOI:** 10.2174/1745017902117010264

**Published:** 2021-12-31

**Authors:** Emanuela Morelli, Olga Mulas, Giovanni Caocci

**Affiliations:** 1 Hematology and CTMO, Businco Hospital, ARNAS “G. Brotzu”, Cagliari, Italy

**Keywords:** Physician, Patient, Communication, Acute myeloid leukemia, Myelodysplastic syndrome, Quality of life

## Abstract

**Introduction:**

An effective communication is an integral part of the patient-physician relationship. Lack of a healthy patient-physician relationship leads to a lower level of patient satisfaction, scarce understanding of interventions and poor adherence to treatment regimes. Patients need to be involved in the therapeutic process and the assessment of risks and perspectives of the illness in order to better evaluate their options. Physicians, in turn, must convey and communicate information clearly in order to avoid misunderstandings and consequently poor medical care. The patient-physician relationship in cancer care is extremely delicate due to the complexity of the disease. In cancer diagnosis, the physician must adopt a communicative approach that considers the psychosocial factors, needs and patient’s preferences for information,which in turn all contribute to affect clinical outcomes.

**Search Strategy and Methods:**

This review was conducted using the Preferred Reporting Items for Systematic and Meta-analyses (PRISMA) statement. We included studies on the importance of physician-patient communication in Acute Myeloid Leukaemia and Myelodysplastic Syndrome care. We searched PubMed, Web of Sciences, Scopus, Google scholar for studies published from December 1 ^st^ , 2020 up to March 1 ^st^ , 2021. Using MeSH headings, we search for the terms “Physician and patient communication AND Acute Myeloid leukemia” or “Myelodysplastic syndrome” or “Doctor” or “Clinician”, as well as variations thereof .

**Purpose of the Review:**

This review examines the progress in communication research between patient and physician and focuses on the impact of communication styles on patient-physician relationshipin hematologic cancers, including Acute Myeloid Leukaemia and Myelodysplastic Syndromes.

## 1 INTRODUCTION

Effective physician-patient communication is of central importance in the field of care and essential to improve clinical encounters in an everyday medical context. In the past, the role of the physician was authoritative and paternalistic. Recently however, medical practice has changed to a more participative relationship between patient and physician with the purpose of creating an alliance that meets the needs of both [1, 2].What defines a patient? The patient is not just an assemblage of organs, symptoms, and emotions but a human being who seeks comprehension and trust. What makes a good physician? A good physician possesses not only an extensive knowledge of symptoms of illness, diagnosis and treatments but also the ability to convey information clearly with good communicative skills to avoid misunderstanding and consequently poor compliance [3]. Two forms of communication have been distinguished in daily medical practice: verbal and non-verbal that contain a message perceived either empathic or detached by the patients [4]. Kaplan *et al* in 1996 administrated a self-reported questionnaire to patients in order to characterize physician's care styles. They demonstrated that a physician's propensity to involve patients in diagnostic and treatment decisions is influenced by their medical background, training and autonomy, suggesting that the physician’s professional and medical satisfaction can influence their communication style [5]. As the medical model has evolved from paternalism to individualism, exchange of information became a dominant communication model, for example shared decision making and the patient-centered communication model. The “shared decision-making model” (SDM), a method where clinicians and patients make decisions together, has positive effects on patient and physician’s satisfaction and is associated with increased knowledge, less decisional hesitancy, and reduced anxiety regarding treatment [6, 7]. Patients need to be involved in the therapeutic process and the assessment of risks and perspectives of the illness in order to better evaluate their options [2]. In particular, the efficacy of SDM practice seems to be more manifest in chronic conditions when patients need routine follow-ups. More importantly, SDM was reported to be associated with improved health outcomes [8].

Patient-centered practices such as the SDM model impact patients' health especially through consens us between the patient and the physician based on shared goals and purposes [8, 9]. As described by Rathert C. *et al*., a collaborative relationship is more likely to lead to excellent clinical outcomes [10]. To conduct an effective patient-centered practice, the physician should develop a compassionate communication style in order to explore the emotional response of the patient [11, 12]. For the physician to understand the patient's perspective requires exploring the patient's feelings, and expectations [13]. Among the non-medical factors associated with increased psychological distress, limited health literacy (LHL) must be taken into account by the physician, as it may limit physician-patient communication [14]. Health literacy (HL) is defined as the skill of individuals to obtain and understand basic health information and thus make health decisions. A limited comprehension of health information would reduce the possibility of the patient to consider their treatment options. The capacity of patients to interpret and understand medical documentation depends ontheir education, culture, and language level [15], suggesting that cultural diversity of patients has to be addressed when establishing a patient-physician relationship [16].

The physician before diagnosis should understand the patient's prior medical knowledge and preferences [13]. However, a variety of studies have shown that physicians and patients have different views regarding what might be an efficient communication and what the patients expect or desire to know about their disease condition [12, 17]. Physicians’ perceptions are frequently inconsistent with patients’ stated preferences [18]. Perron NJ *et al*., explored patients expectations in multicultural contexts and physicians were found to be generally poor at identifying patients’ preferences, making cross-cultural communication even more complex [19]. Although, it was reported that about 70% of the patients prefer a type of patient-centered communication, cultural gaps can constitute a limitation [13] (Table **1**).

## 2 IMPACT OF PATIENT-CLINICIAN COMMUNICATION IN CANCER CARE

**Fig. (1):**
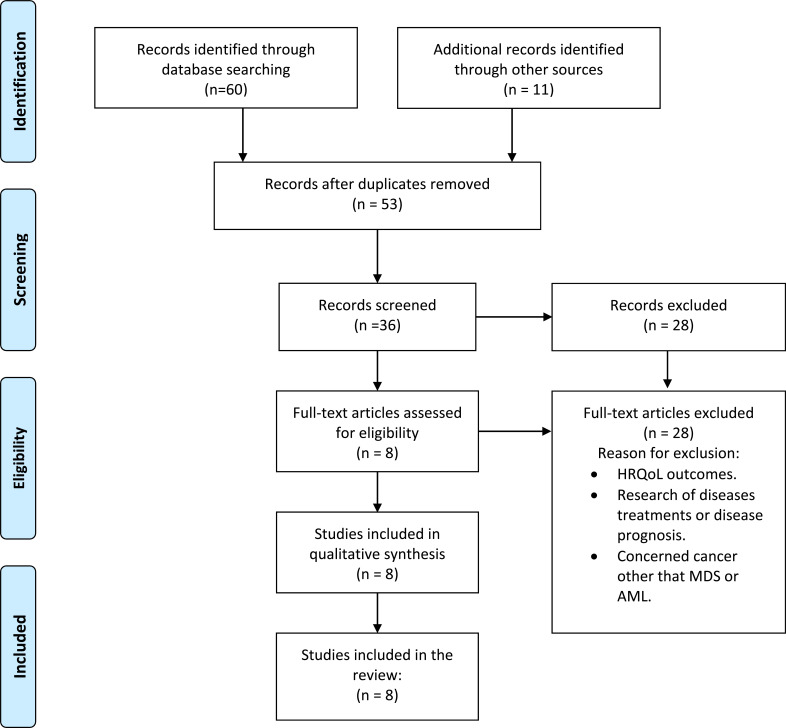
Study flow diagram. The databases searched were PubMed, Web of Sciences, Scopus, Google scholar. The results were defined using the Preferred Reporting Items for Systematic and Meta-analyses (PRISMA) statement to identify, select, and determine eligibility of papers for inclusion in the study.

Over the past years, prioritizing cancer patients' needs has become an important goal. Cancer patients more frequently seek for further information about their diagnosis and treatments, especially the younger ones [20]. Amongst cancer patients, LHL has been associated with poor health quality of life (HQoL), increased anxiety and more mental distress [16, 21]. Therefore, it is the goal of the physician, to promote an inter-personal connection; to share medical information and to put the patients in the position of being able of choosing amongst available treatments [22]. Especially for the advanced cancer population, it is challenging to make a prognosis to predict the patient’s expectation for care [23]. In general, older patients are less likely to want prognosis information probably due to the complexity of the disease or a status of anxiety [24]. Most physicians experience difficulty communicating prognosis information to patients [25]. Yet, patients rank physician communication manner as highly important, and patients prefer sensitivity and honesty from their physicians [25-28]. Physicians, in contrast rank patient-physician communication as the least important in the relationship [17]. Nevertheless, other studies have demonstrated that competence aside, addressing patients’ emotional needs does impact on patient satisfaction more [29]. “*The patient will never care how much you know, until they know how much you care*” [30]. The patient-physician relationship in cancer care is extremely delicate and sensitive due to the emotional factors associated with the disease. A recent review, based on the experiences of patients, families and experts, setup a guideline that represents high level recommendations with the purpose of optimizing patient-physician relationship and well-being in cancer care. The results of the analysis identified a core of central communication skills such as: to discuss end-of-life care, meet the needs of the patient and facilitate the communication with the family, discuss cost of care, consider cultural gaps *etc* [31]. Here, we examined patient-physician relationships and communication styles in light of research evidence focusing on hematologic cancers, including Acute Myeloid Leukaemia and Myelodysplastic Syndromes (Fig. **1**).

## 3 PATIENT-PHYSICIAN COMMUNICATION IN ACUTE MYELOID LEUKAEMIA

Acute myeloid leukaemia (AML) is unusual compared with other cancer forms, in its prognostic variability, treatment intensity choices and chances forcures, even if new drugs have recently emerged [32, 33]. Specifically, AML is a malignant disease of uncontrolled accumulation of undifferentiated myeloid precursor cells, most commonly in the bone marrow [BM] that leads to BM failure and death. Without treatment, AML progresses rapidly, in fact survival is measured in days to weeks. A study based on the Global Burden of Disease 2017 database revealed that the incidence of AML gradually increased in most countries from 1990 to 2017 and, in line with other studies, found that males and elder persons have a higher risk of developing AML [34, 35]. In fact, the risk of developing AML increases with age, with over 70% of new AML cases being diagnosed in adults of> 55 years, with a 5-years survival rate of 3-8% in patients over 60 years [20, 32, 36, 37].

Walsh E.H. *et al*, in 2019 conducted research that explored the degree of concordance between the patient experience of symptoms as reported respectively by the AML patients themselves or by the physicians. Furthermore, they examined how the degree of discordance impacted on the health-related quality of life (HRQoL) of the patient. Agreement on the individual symptoms varied considerably. The results described the lowest level of agreement for appetite loss, reported by 74% of AML patients compared with only 11% of physicians, and the highest agreement for fever with 12% of AML patient self-reporting these symptoms and 7% reported by the physicians [37]. This study has demonstrated not only a substantial disagreement between patients and physician-reported symptoms, but also that the level of discordance negatively influences the HRQoL of AML patients.

Due to the nature of AML disease and its rapid progression the patient is often forced to make quick decisions under a lot of pressure about available treatments and sometimes without a transparent and empathic behaviour from the physician. Physicians working with these patients are encouraged to work together with patients, their families, and also with the medical team to reach the best results. A qualitative study has described the way that patients newly diagnosed with AML experience medical and social challenges. Especially, researchers have focused on the emotional reaction of AML patients to the diagnosis, their information needs and treatment planning decisions. It was reported that the patients newly diagnosed with AML criticised the poor physician communicationand complained about an inconsistency between information provided and information required [32]. The results of the study,together with the complex medical information the AML patients have to deal with, explains why the majority of patients prefera more passive or collaborative decision-making approach as described by Yogaparan *et al*. [38]. How do patients perceive the potential risk and benefits of the therapy? Is it in accordance with the physician’s perception? El-Jawahri *et al*., performed a prospective analysis to examine the perception of older patients with AML regarding the risks and benefits and observed that the degree of prognostic misperception was prominent. The patients often overestimate the risk of treatment-related mortality compared to their oncologist. Similar discordances were observed with regard to the chance of cure: 90% of patients thought they were very likely to be cured of their leukaemia, whilst only 31% of the oncologists reported the same chance. Patients’ misperception may hinder a clear evaluation of the risk or benefit of the therapy supporting the importance of communication skills as tools to enhance patient knowledge and therefore the understanding of the impact the interventions can have on their health [39].

## 4 PATIENT-PHYSICIAN COMMUNICATION IN MYELODYSPLASTIC SYNDROME (MDS)

The myelodysplastic syndromes (MDSs) area very heterogeneous potentially life-threatening myeloid disorders characterised by peripheral blood cytopenias and a tendency to progress to AML [40]. MDS occurs more often in older males and in individuals previously exposed to cytotoxic therapies such as chemotherapy or radiation therapy [41]. Since 2001, cases of MDSs have been tracked by cancer registries. In the United States, the incidence of MDS is reported to be 4.9 per 100,000 population/year for 2007–2011 [42]. Studies conducted in Europe, specifically in the Düsseldorf Registry described an incidence of 4.15 cases [43]; a Swiss study showed an incidence of 3.6 cases per million [44]; a Swedish study described 1,329 patients with MDS, corresponding to 2.9 cases per 100,000 population/year. The average age was 71, however in all registries the incidence gradually increases with age, making MDS one of the most common blood cancers in the elderly population [45]. Due to cytopenia and other symptoms such as fatigue, pain and anxiety, MDS can have an impact on a patient’s daily life and HRQoL [46]. In fact, a diagnosis of MDS can trigger psychological, social and economic stresson patients, besides the difficulty of understanding the disease and the aim of the treatment. In most patients with MDS,the fear for a possible progression of the disease to AML causes them often to experience a lack of hope and a feeling of anger towards the physician [47]. Depression occurs in 10%-22% of the patients with cancer and patients with MDS also have higher risk of depression [47, 48].

Sekeres *et al*. in 2011 demonstrated that MDS patients have a limited understanding of their disease, prognosis and treatment options [49]. Interestingly, a survey performed in patients with MDS suggests that patient understanding of treatment goals and prognosis is often limited, with a third of the patients reporting that prognosis was not discussed with their physician. In line with this finding, anotherstudy performed with MDS patients described a poor patient understanding of treatment goals and a lack of time and discussion with the physician [50]. Further studies have evaluated physicians’ perception of the health status of their patients and their desire to be involved in decisions showing that the physician did not adequately identify the patient’s preferences [18]. However, further findings have pointed out that MDS patients with severe health conditions prefer not to participate intreatment decisions and to leave treatment decisions to their physician [23]. In patients with MDS a lack of information received and understood may provoke feelings of distress.A cross sectional survey, conducted in France and Australia, showed that inadequate information exchange and communication with physicians were associated with higher distress reinforcing the importance of effective communication between patients and physicians [51]. Often medical routine examination is conducted at the expense of emotional and psychological status of the patient. Physicians should encourage open communication and facilitate exchange of psychological and emotional needs in order to support the patient well-being (Table **2**).

## CONCLUSION

Recognising patient-physician communication as a major patient-physician component in the relationship has become of fundamental importance. As changes have occurred in medical policy, including the introduction of consensus statements and guidelines, it became even more significant to progress further in the dyadic physician–patient interaction. Most patients complain about the detached attitude of the physician to their requests leading to misunderstandings and dissatisfaction. Patients want more information about their condition and treatment outcomes, more information on the side-effects of the treatments and prefer to be engaged in the therapeutic decision [52]. Several studies have indicated that effective patient-physician communication is related to improved adherence to treatment regimens, better decision making, less medical malpractice claims, and increased satisfaction [3, 22, 53]. Opting for an open, responsive, and mutual decision-making relationship is not only to the advantage of the patients but also to the physicians. In fact physicians, who practice relationship-centered care with patients, have been found to be more satisfied with their profession [54]. An effective patient-physician communication, particularly in cancer care, helps physicians in delivering bad news. Accordingly, cancer communication research indicates that the manner in which clinicians address disease issues plays a key role in the continuing adjustment of patients with cancer [4, 55-58]. Thus, it is clear that communication skills, including delivering bad news, should be taught and it has been demonstrated that these competencies can be learnt by the physicians [59]. As a consequence, over the last years, health care organizations have implemented communication skills training for physicians to enhance patients’ satisfaction with the quality of care received [4]. In the physician-patient dyad, there also emerged the current need to consider the new online healthcare community and their impact on information processing, health decisions and behaviour, and the quality of life of the patient. The extensive use of information and communication technology as a source of health information raised concerns about its effects on the physician-patient relationship. Concerns include access of multiple information sources that can result in mistaken self-evaluation and self-treatment by the patient and disagreements with the physician’s practice [60, 61]. However,studies have reported that for some physicians exposure to online health information can have a positive impact on a patient’s sense of confidence and control during interactions, as well as improving the patient’s understanding of medical heath issues [62, 63]. Given these considerations, the experience and physician’s encouragement are even more necessary to help the patient to interpret and apply this information. As studies have indicated, efficient communication and educational resources can increase understanding of disease and achieve better results, including improved treatment outcomes. Physicians have to takea new approach to ensure; i) that the patients truly understand, taking the time when needed to counsel and listen to patients; ii] to remain committed to their work; iii) to improve collaboration and coordination with the medical team [64, 65]. In conclusion, the research on physician-patient communication and interaction still need further analysis and requires implementation of methods and intervention models that can define the rules to regulate the physician-patient relationship.

## Figures and Tables

**Table 1:** Summary of good physician communicative skills.

**Good Interpersonal and Effective Communicative Skills**
=> Demonstrate caring and respectful behaviors=> Patients need is to be involved in the therapeutic process.=> Take the time when needed to counsel and listen to patients.=> Provide medical information using effective instructions to avoid misunderstanding.=> Check accuracy of patient’s understanding.=> Help the patient to make decision about the treatment options based on their preferences.=> Address the patient’s perspective on the illness.=> Consider psychosocial factors of the patient, cultural gap and language limits.=> Consider the expansion of new communication practices and technologies and their impact on information processing, health decisions and behaviour.
**Preferred Communication Style**
=> Acceptance=> Empathy=> Frankness=> Simplicity=> Honesty

**Table 2:** Included studies.

**Authors**	**Title**	**Journal**	**Methodology**	**Partecipants**	**Outcomes**
Youssoufa M. Ousseine; *et al*. 2018	Association between health literacy, communication and psychological distress among myelodysplastic syndromes patients	Lukemia Research	Cross-sectional survey. Self- administrated questionnaire	280 MDS patients; 154 French and 126 Austrialian; median age 69.5 years.	Inadequate functional Health Literacy was associated with higher global distress particularly in MDS patients due to the heterogeneity of the symdrome.
Mikkael A Sekeres: *et al*. 2011	Perceptions of disease state, treatment outcomes, and prognosis among patients with myelodysplastic syndromes: results from an internet-based survey	The Oncologist	Internet-based survey	3.131 patients were invited to participate; 361 completed the survey.	The study shows that patients with MDS have a limited understanding of their disease, prognosis and treatment goals.
B. Douglas Smith, MD; 2015	Myelodysplastic Syndromes: Challenges to Improving Patient and Caregiver Satisfaction	The American Journal of Medicine		The sample included 358 patients. The median age: 65 years old.	An effective physician and patients communication is demonstrated to impact on patients exploring behavior, potentially new curative treatments and clinical trials.
L. Elise Horvath Walsh; *et al*. 2019	Real-World Impact of Physician and Patient Discordance on Health-Related Quality of Life in US Patients with Acute Myeloid Leukemia	Oncology and Therapy	Patient self-completion (PSC) form	61 physicians included 457 AML patients: 82 AML patients agreed to complete PSC from. 44% were female; the median age was 60 years old.	The study reports a substantial discordance between patients-reported and physicians-estimated symptoms.
Lagadinou D. Eleni; *et al*. 2010	Challenges in treating older patients with Acute Myeloid Leukemia	Journal of Oncology	Review article		This paper reviews the most optimal treatment strategies, risks and benefits for elderly AML patients. Results in allogeneic transplantation are very promising.
Lone S Friis; *et al*. 2003	The patient's perspective: a qualitative study of Acute Myeloid Leukemia patients' need for information and their information-seeking behavior	Supportive Care in Cancer	In-depth ethnographic interviews	A total of 21 AML patients; 11 female and 10 male. Average age of the sample was: <50 (9); 50–70 (6) >70 (6) years old.	This study demonstrates that the expressed attitude of the AML patients regarding the need for medical information is discordant with patient's real information-seeking behavior.
Areej El-Jawahri; *et al*. 2019	Patient-Clinician Discordance in Perceptions of Treatment Risks and Benefits in Older Patients with Acute Myeloid Leukemia	The Oncologist	Longitudinal study	Newly diagnosed; 100 AML patients and 11 oncologists. Older AML patients over the age of 60 years old.	Older AML patients overestimate both the risks and benefits of treatment and have misperceptions about their prognosis.
Thomas W LeBlanc; *et al*. 2017	Patient experiences of Acute Myeloid Leukemia: A qualitative study about diagnosis, illness understanding, and treatment decision-making	Psychooncology	Semi-structured qualitative interviews	32 AML patients completed the interview. Average age of 60 years old or older.	This paper underlines the need for targeted interventions to improve AML patients' understanding of the disease and treatment options.
